# Integrating Sexual and Reproductive Health Services Within HIV Services: WHO Guidance

**DOI:** 10.3389/fgwh.2021.735281

**Published:** 2021-10-01

**Authors:** Nathan Ford, Morkor Newman, Sarai Malumo, Lastone Chitembo, Mary E. Gaffield

**Affiliations:** ^1^Department of HIV, Viral Hepatitis and STIs, World Health Organization, Geneva, Switzerland; ^2^Department of Reproductive and Women's Health, World Health Organization, Lusaka, Zambia; ^3^Department of HIV, Viral Hepatitis and STIs, World Health Organization, Lusaka, Zambia; ^4^Human Reproduction Programme (HRP), World Health Organization, Geneve, Switzerland

**Keywords:** contraception, sexual and reproductive health, HIV, guidelines, family planning

## Abstract

Among the 1.9 billion women of reproductive age worldwide in 2019, 1.1 billion need family planning and 270 million have an unmet need for contraception. For women and adolescent girls living with human immunodeficiency virus (HIV), using effective contraception reduces the mother-to-child transmission of HIV by preventing unintended pregnancies and enabling the planning and safer conception of desired pregnancies with optimal maternal and child health outcomes. The World Health Organization (WHO) recommends that sexual and reproductive health services, including contraception, may be integrated within HIV services. Integration is associated with increased offers and uptake of sexual and reproductive health services, including contraception, which is likely to result in improved downstream clinical outcomes. Integrating HIV and sexual and reproductive health services has been found to improve access, the quality of antenatal care and nurse productivity while reducing stigma and without compromising uptake of care. Research is encouraged to identify approaches to integration that lead to better uptake of sexual and reproductive health services, including contraception. Implementation research is encouraged to evaluate different strategies of integration in different health systems and social contexts; such research should include providing contraception, including long-acting contraception, in the context of less frequent clinical and ART refill visits.

## Introduction

Among the 1.9 billion women of reproductive age (15–49 years old) worldwide in 2019, 1.1 billion need family planning and 270 million have an unmet need for contraception. Across regions, evidence indicates that sex workers are at higher risk of both acquiring human immunodeficiency virus (HIV) and having an unintended pregnancy, have a greater unmet need for contraception than the general population, rely excessively on using condoms alone instead of the recommended dual protection, and face stigma from health care providers (1–5). The proportion of the need for sexual and reproductive health including contraception services that was satisfied by modern methods was 76% globally in 2019, but this fell to <50% in western and central Africa. By enabling women and adolescent girls to exercise their right to choose and control whether to have children and when and how many, use of voluntary, effective contraception promotes positive educational and economic outcomes for women and girls and is key to achieving gender equality, empowering women and reducing poverty. Using contraception also leads to improved infant and child health outcomes by preventing morbidity and mortality related to unintended pregnancy. For women and adolescent girls living with HIV, using effective contraception reduces the mother-to-child transmission of HIV by preventing unintended pregnancies and enabling the planning and safer conception of desired pregnancies with optimal maternal and child health outcomes (6).

The World Health Organization (WHO) emphasizes the importance of linking sexual and reproductive health and rights and HIV for adolescent girls and young women (7). Since women living with HIV face unique challenges and human rights violations related to their sexuality and reproduction within their families and communities and from the health-care institutions in which they seek care, particular emphasis is placed on creating an enabling environment to support more effective health interventions and better health outcomes (8).

WHO recommends the integration of HIV services, including HIV testing services (HTS), with a range of other relevant clinical services, such as those for TB, maternal and child health, sexual and reproductive health, harm reduction programmes for people who inject drugs and, in priority countries, voluntary medical male circumcision (VMMC) programmes (9). Integration has been found to lead to a range of benefits across disease areas (10–12). In 2016, WHO made a conditional recommendation that sexually transmitted infection and family planning services can be integrated within HIV care settings (9). WHO also recommends that, with the exception of advanced HIV disease, women living with HIV can generally use any contraceptive method (13).

## Updated WHO Guidance

Since 2016, additional evidence has been published supporting the integration of sexual and reproductive health and HIV. A systematic review published in 2019 that considered linkage and integration found that the proportion of women receiving an HIV test during the study period ranged from 35 to 99% for integrated services and from 20 to 95% for non-integrated services or services integrated at a lower level (14); the review summarized findings from several studies that included adolescent girls and young women. The proportion of women accessing HIV services using contraception ranged from 54 to 80% for integrated services and from 10 to 83% for non-integrated services (14). Integrating HIV testing services with sexual and reproductive health services is feasible and has potential for positive joint outcomes. The review included six studies—one cluster randomized trial carried out in Uganda and five non-randomized cluster trials carried out in Kenya, eSwatini and the United States of America. A recent landscape analysis of 12 programmes offering HIV testing services within family planning found that integration of HIV testing services within family planning/SRH services was highly variable, with limited information reported about how integration was implemented (15).

In 2020, WHO convened a guideline development group to formulate updated recommendations for HIV service deliver. As part of this, WHO commissioned a systematic evidence review to inform the potential updating of recommendations on integration of HIV and sexual and reproductive services.

## Evidence Review

A systematic review identified two studies which reported an increase in sexual and reproductive health service uptake, including contraception, associated with integration; one study found an increase in the uptake of dual contraceptive methods. In the study that reported dual method use, the proportion of women using dual methods during the study period was 34% for integrated services and 0% for non-integrated services (16). The overall certainty of evidence for all outcomes was very low, and the available evidence is limited. In the other direction, another systematic review of 14 studies found that integrating family planning into HIV care and treatment settings was associated with higher levels of use and knowledge of modern methods of contraception among women living with HIV (17).

Considering this evidence, the guideline development group recommended that sexual and reproductive health services, including contraception, may be integrated within HIV services. This was a conditional recommendation, based on the very-low certainty evidence available to support the recommendation.

Integration is associated with increased offers and uptake of sexual and reproductive health services, including contraception, which is likely to result in improved downstream clinical outcomes. One concern about integration is that tasking providers with too many services may reduce the quality of these services. However, it has been reported that integration can yield positive effects on service quality as well as client outcomes for contraceptive use, ART in pregnancy and HIV testing (18). Integrating HIV and sexual and reproductive health services has been found to improve access, the quality of antenatal care and nurse productivity while reducing stigma and without compromising uptake of care (19). Overall, the Guideline Development Group considered that the benefits likely outweighed any possible harm.

In terms of feasibility and cost, a study from Zambia found that integrated HIV testing and counseling and voluntary medical male circumcision services could be provided at a lower cost per client than segmented, vertical provision (20). A study from Kenya (21) found decreased consultation times when services were integrated (10 vs. 30 min); another study from Kenya (22) highlighted the need for sustained systems and health-care worker support over time. Integration may lead to increases in service efficiency, but this is likely to be highly context dependent (21, 23, 24).

## Equity, Acceptability and Implementation Considerations

A survey conducted among health-care providers and clients in support of these guidelines found that more than 90% of respondents considered that integration is important and feasible. Integration may improve access to sexual and reproductive health services, including contraception, among key populations. A study from Kenya found that access to sexual and reproductive health services, including contraception, for women who inject drugs can be improved by integrating contraceptive and other sexual and reproductive health interventions into existing outreach-based HIV prevention and harm-reduction programmes (25). Another study among female sex workers in Kenya found that integration improved access to the use of non-condom contraception methods, which is important for this group, who may have difficulty negotiating condom use (26). Integration also has the potential to reduce stigma. A survey of health-care providers in South Africa found that they considered integration important for reducing stigma and increasing access to and improving the quality of care (26).

Implementing comprehensive and integrated sexual and reproductive health and rights and HIV programmes to meet the health needs and rights of the diverse group of women living with HIV requires that interventions be put in place to overcome barriers to service uptake, use and continued engagement. In all epidemic contexts, these barriers arise at the individual, interpersonal, community and societal levels. They may include challenges such as social exclusion and marginalization, criminalization, stigma, gender inequality, and gender-based violence; WHO strongly recommends that care for women experiencing intimate partner violence and sexual assault, as much as possible, be integrated into existing health services (27). Strategies are needed across health system building blocks to improve the accessibility, acceptability, affordability, uptake, equitable coverage, quality, effectiveness and efficiency of services for women living with HIV. If left unaddressed, such barriers undermine health interventions and the sexual and reproductive health and rights of women living with HIV (27).

Experience from field work has shown that the lower the level of service delivery, the greater the extent of integration, as there are fewer dedicated health worker to provide the service. In many places, integration is limited by inadequate infrastructure and commodities which cannot adequately respond to the high client load. A focus on improving investment in the overall health system will be important to support the integration of sexual and reproductive health services, including contraception and HIV services. Laws and policy barriers to accessing sexual and reproductive health services, including for adolescents, need to be addressed. Although this applies to any integration effort, it is especially important since sexual and reproductive health programmes have historically been implemented as established vertical programmes within health systems. There is limited data on the extent to which HIV testing services have been integrated into family planning services and whether such integration has had a positive effect on uptake of either services (15).

Training on human sexuality may facilitate greater understanding of sexually diverse communities, particularly those identifying as lesbian, gay, bi-sexual, transgender, queer or intersex (LGBTQI), as well as adolescents and young people seeking accurate SRHR information and services (28).

Since an increasing proportion of people living with HIV are receiving their HIV treatment through a differentiated service delivery model with extended ART refills and less frequent clinical visits, aligning the provision of sexual and reproductive health services, including contraception commodities—WHO recommends providing 1 year of oral contraception and supports community delivery and self-management—with differentiated service delivery for HIV treatment models should be considered.

Careful planning and coordination are important for both programme management and service delivery, including establishing integrated data systems and providing consistent cross-training of health-care providers. Political will significant coordination, collaboration and integration across disease programmes are important (29, 30).

## Research Gaps

Conditional recommendations are widely taken up on policy (31). The issuing of a conditional, as opposed to a strong, recommendation implies that for patients the majority of people would want the recommendation, but many would not; for policy-makers, it implies that there is a need for debate and the involvement of stakeholders in order to implement the recommendation. Conditional recommendations are frequently made when the available evidence to support the course of action is limited and of low certainty. This provides a clear indication that further research is needed (32).

The evidence supporting approaches to integrating sexual and reproductive health services, including a range of contraception, with HIV services is limited (15). Evidence from a robust research study setting (the ECHO trial) found that HIV and STI incidence rates were very high in a population accessing family planning, highlighting that urgent efforts to monitor and test intervention efforts in the public sector are needed (33). Research is encouraged to identify approaches to integration that lead to better uptake of sexual and reproductive health services, including contraception; such research should also consider integrating cervical cancer screening and vaccination. Implementation research is encouraged to evaluate different strategies of integration in different health systems and social contexts; such research should include providing contraception, including long-acting contraception, in the context of less frequent clinical and ART refill visits.

## Conclusions

There is a pressing unmet need for sexual and reproductive health services, including contraception. The updated guidance from WHO aims to support increased access to services through integration. There are many evidence gaps and high-quality research is needed.

## Author Contributions

NF drafted the article. All authors contributed to critical revision and approved the final version.

## Funding

This work was supported by a grant from the Bill & Melinda Gates Foundation.

## Conflict of Interest

The authors declare that the research was conducted in the absence of any commercial or financial relationships that could be construed as a potential conflict of interest.

## Publisher's Note

All claims expressed in this article are solely those of the authors and do not necessarily represent those of their affiliated organizations, or those of the publisher, the editors and the reviewers. Any product that may be evaluated in this article, or claim that may be made by its manufacturer, is not guaranteed or endorsed by the publisher.
